# Alcohol consumption as a risk factor for sarcopenia - a meta-analysis

**DOI:** 10.1186/s12877-016-0270-x

**Published:** 2016-05-11

**Authors:** Michal Steffl, Richard W. Bohannon, Miroslav Petr, Eva Kohlikova, Iva Holmerova

**Affiliations:** Department of Physiology and Biochemistry, Faculty of Physical Education and Sport, Charles University Prague, Prague, Czech Republic; Department of Physical Therapy, College of Pharmacy and Health Sciences, Campbell University, Buies Creek, NC USA; Centre of Gerontology, Faculty of Humanities, Charles University Prague, Prague, Czech Republic

**Keywords:** Aging, Sarcopenia, Alcohol

## Abstract

**Background:**

Sarcopenia, a loss of muscle strength and mass, has serious implications for older adults. Some risk factors for sarcopenia are well established. The role of other factors such as alcohol consumption is less certain. The main aim of this study was to explore the relationship between sarcopenia and alcohol consumption in people over 65 years old.

**Methods:**

Four electronic databases were searched to identify potentially relevant papers. Demographics and information on sarcopenia and alcohol consumption were extracted from relevant papers. The relationship between sarcopenia and alcohol consumption was described using odds ratios (ORs).

**Results:**

Of 214 papers identified as potentially relevant, 13 were ultimately included in the meta-analyses. The papers provided data from 13,155 participants. The OR (95 % CI) for sarcopenia among alcohol drinkers was 0.67 (0.54–0.83) for males, 0.89 (0.73–1.08) for females, and 0.77 (0.67–0.88) for the overall population.

**Conclusions:**

The results of this meta-analysis do not support alcohol consumption as a risk factor for sarcopenia.

## Background

Although old age is not a disease, many diseases and syndromes are more prevalent among older adults. As the proportion of older adults is increasing, an understanding of age-related diseases and syndromes is critical. One syndrome particularly common among older adults is sarcopenia [1]. Described by the European Working Group on Sarcopenia in Older People (EWGSOP) as a loss of muscle mass and strength and decreased physical performance, sarcopenia is associated with untoward outcomes such as disability, falls, and mortality [2]. Although deterioration of muscle and functional limitations have many causes and accompany natural aging, they could be accelerated by modifiable behavioral factors such as inactivity, undernutrition, smoking, and alcohol consumption. The possible role of alcohol consumption is of particular interest as it is associated with a number of pathologies, including alcoholic liver disease, pancreatic disease, neurological problems, cancer, and immunosuppression [3]. Ethanol impairs skeletal muscle protein synthesis and muscle autophagy is increased by ethanol exposure [4]. Alcohol consumption is associated with cachexia [5]. Whether, however, alcohol consumption is a risk factor for sarcopenia is unclear. Although individual risk factors do not work independently, the role of alcohol consumption as a risk factor for sarcopenia should be clarified. The main objective of this study was to explore relationships between sarcopenia and alcohol consumption in people over 65 years of age. Following the recommendation of the Cochrane Handbook for Systematic Reviews [6], we pursued this objective by calculating odds ratios ORs. The OR compares the relative odds of an outcome occurring (in our case sarcopenia) based on exposure to a variable of interest (in our case alcohol). In our study an OR greater than 1 would suggest that sarcopenia is associated with higher odds of developing sarcopenia and an OR less than 1 would suggest that sarcopenia is associated with lower odds of having sarcopenia.

## Methods

This review included cross-sectional studies and data from baselines of longitudinal cohort studies. For the sake of meta-analyses, all data were treated as if from a case–control study- where sarcopenia was considered as the case and alcohol consumption represented the exposure. Participants who were included in those studies were people over 65 years of age who lived primarily in their own homes in the community. Ethical approval and consent from participants were declared in all the studies which were analyzed.

### Search methods for identification of studies

A 2-stage systematic review of the literature was used to identify appropriate papers. Stage 1 involved searches of four electronic databases: PubMed, Web of Knowledge, EBSCO and Sciencedirect. The same search stream was used in all databases (Table 1). Stage 2 involved examining cited references of relevant studies and review papers identified during stage 1.

**Table 1:** Search strings employed and yield associated with searches of four electronic databases

Database (yield)	Search terms
PubMed	Search (sarcopenia [Title] OR “low muscle mass” [Title] OR “lean body mass” [Title] OR “skeletal muscle mass” [Title]) AND (alcohol* OR “alcohol drink*” OR “alcohol consumption” OR ethanol)
Web of Knowledge	TITLE: (sarcopenia OR “low muscle mass” OR “lean body mass” OR “skeletal muscle mass”) AND TOPIC: (alcohol* OR “alcohol drink*” OR “alcohol consumption” OR ethanol)
EBSCO	TI (sarcopenia OR “low muscle mass” OR “lean body mass” OR “skeletal muscle mass”) AND TX (alcohol* OR “alcohol drink*” OR “alcohol consumption” OR ethanol)
Sciencedirect	TITLE (sarcopenia OR “low muscle mass” OR “lean body mass” OR “skeletal muscle mass”) and (alcohol* OR “alcohol drink*” OR “alcohol consumption” OR ethanol)

### Data collection and analysis

All full-text articles were systematically examined for inclusion or exclusion. The primary criterion for including the studies in the meta-analyses was that they transparently presented data regarding alcohol consumption by both sarcopenic and nonsarcpenic individuals. Papers containing duplicate data from a sample of participants were excluded so that data from individual participants was used only once. Acquired outcome and exposure data were dichotomized with the counts for the two variable categories entered into 2 × 2 contingency tables. In some studies, where the sarcopenic status was divided into three categories - non sarcopenia, moderate sarcopenia and severe sarcopenia - the dual model was used; non sarcopenia and sarcopenia. Alcohol consumption was described inconsistently in studies. So individuals were classified as exposed if they consumed alcohol regardless the period or intensity of alcohol drinking.

### Measures of effect sizes

As previously stated, our meta-analysis was based on OR. The Cochran-Mantel-Haenszel statistical method [7] and DerSimonian and Laird random-effects model [8] were used to calculate OR. The Cochran-Mantel-Haenszel statistical method is based on fixed-model effect values of larger studies in contrast with DerSimonian and Laird random-effects model, which gives relatively the same weight to all the studies in the sample [9]. A test for overall effect and a test for overall average effect for random effects meta-analysis were used to choose which model was better to apply. Additionally, we tested heterogeneity to find out if consolidated studies were consistent in their findings. The I^2^ index was used for this purpose. A rough guide to interpretation of I^2^ is as follows: 0 to 40 %: might not be important; 30 to 60 %: may represent moderate heterogeneity; 50 to 90 %: may represent substantial heterogeneity; 75 to 100 %: considerable heterogeneity [10]. Funnel plots were used for visualizing biases. A funnel plot is a simple scatter plot of the exposure effect estimated from individual studies against some measures of each study’s size or precision [6]. Furthermore, we conducted a sensitivity analysis. That analysis involved removing and returning particular studies to determine the best OR estimate taking into account heterogeneity and the robustness of conclusions. Statistics were carried out in the Review Manager 5.3. Additionally, the illustrative comparative risks were calculated and they were presented in summary of findings table. GRADEprofiler was used to create and manage the summary of findings table.

## Results

### Description of studies

**Fig. 1:**
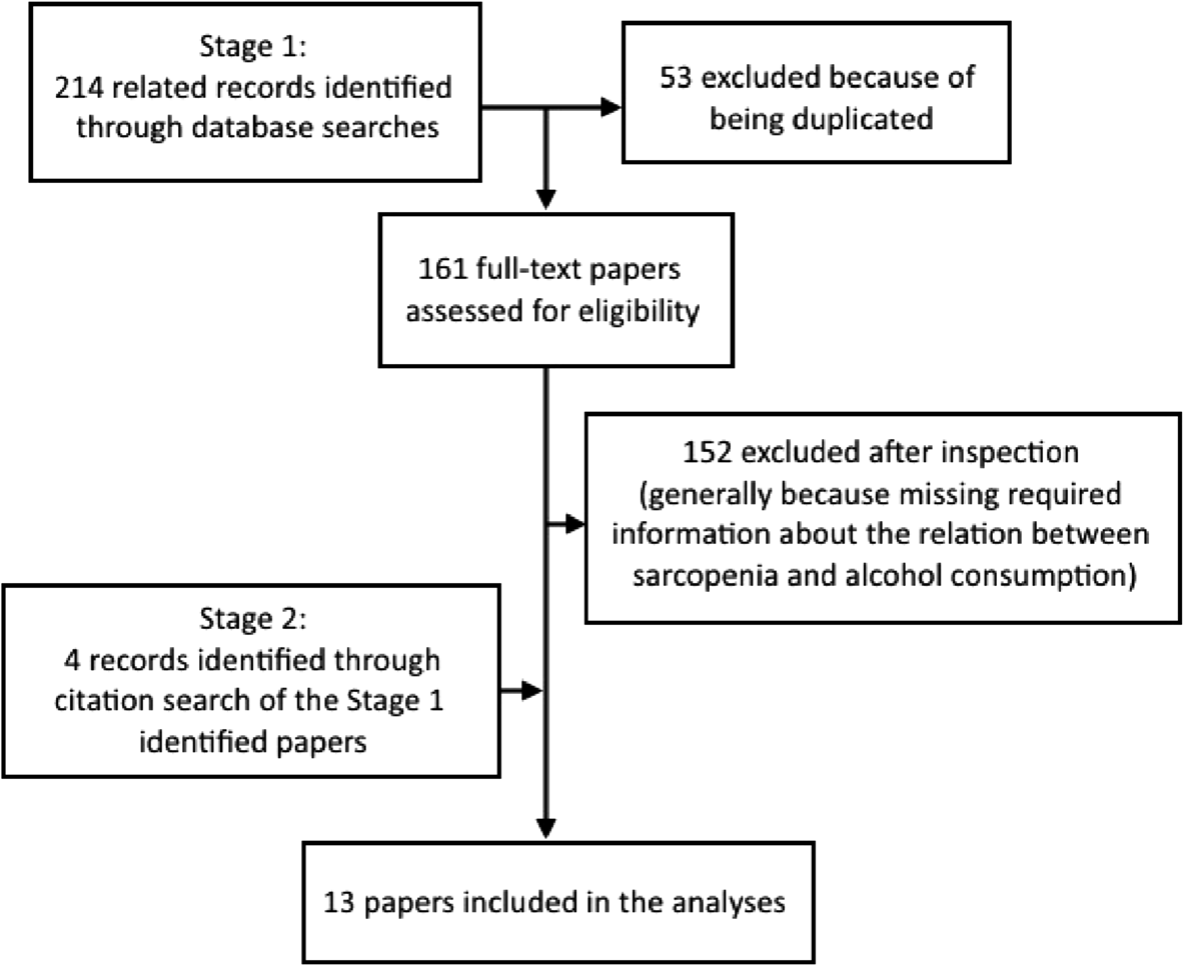
Flow chart indicating how the final sample of papers included in the meta-analysis was established

Figure 1 summarizes the yield of the search process. Of 214 papers identified as potentially relevant by the database searching, 9 were included. An additional 4 papers identified through article reference lists were added. Ultimately, therefore, 13 studies were included in the meta-analyses. The main characteristics of the included studies are presented in Table 2. Data from 13,155 participants were analyzed; these included 5664 males and 7491 females. There was a huge difference in the age of participants in the studies. The mean age ranged from 68.1 (SD = 0.6) years old to 83.8 (SD = 5.9) years old. The prevalence of sarcopenia ranged from 3.8 to 27.2 % and the prevalence of alcohol drinkers extended from 1.2 to 74.6 %.

**Table 2:** Characteristics of studies included in the meta-analyses

Study	Design^a^	Principal Aims
Akune et al. 2013 [21]	Cross-sectional	Investigate the prevalence of sarcopenia using the EWGSOP definition, and clarified the association of sarcopenia with physical performance
Castillo et al. 2003 [23]	Cohort	Examine sarcopenia prevalence and risk factors in community-dwelling men and women who attended a 1988–1992 Rancho Bernardo Study clinic visit
Domiciano et al. 2013 [11]	Cohort	Evaluate the prevalence and risk factors associated with sarcopenia, according to these two criteria in community-dwelling older women
Figueiredo et al. 2013 [27]	Cohort	Evaluate the prevalence and risk factors associated with sarcopenia, based on these two criteria
Landi et al. 2013 [24]	Cohort	Evaluate the impact of sarcopenia on the risk of all-cause death in a population of frail older persons
Lau et al. 2005 [13]	Cross-sectional	Evaluate the prevalence of and risk factors for sarcopenia in elderly Chinese, and to compare these observations with those in white persons
Lin et al. 2013 [28]	Cross-sectional	Determine the prevalence of sarcopenia using the EWGSOP algorithm in a general elderly population in a Taiwanese metropolitan area
Liu et al. 2014 [14]	Cohort	Evaluate the prevalence of sarcopenia and its associative clinical characteristics
Park et al. 2014 [22]	Cross-sectional	Examine whether vitamin D deficiency was positively associated with sarcopenia in a gender-specific manner in adults aged 50 years, independent of other covariates and possible confounders, including body composition, blood tests, including serum parathyroid hormone (PTH) levels, dietary intake, and hormone replacement therapy in women
Sampaio et al. 2014 [29]	Cross-sectional	Examine whether arterial stiffness, measured by the cardio-ankle vascular index (CAVI), is associated with skeletal muscle mass index (SMI) in Japanese community-dwelling older adults.
Silva Alexandre et al. 2014 [20]	Cross-sectional	Examine the prevalence and factors associated with sarcopenia in older residents in São Paulo, Brazil
Volpato et al. 2014 [15]	Cross-sectional	Estimate the prevalence and investigate the clinical correlates of sarcopenia
Wu et al. 2014 [16]	Cross-sectional	Show the prevalence and associated factors of sarcopenia and severe sarcopenia in rural communitydwelling older Taiwanese

^a^As stated by the authors

### Summary of the outcomes

**Fig. 2:**
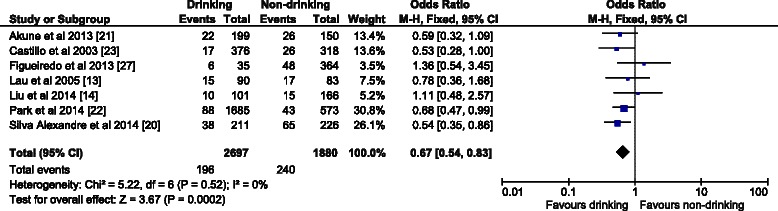
The forest plot of effect sizes for alcohol drinking males

**Fig. 3:**
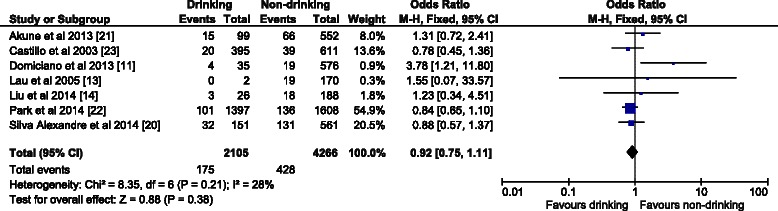
The forest plot of effect sizes for alcohol drinking females

**Fig. 4:**
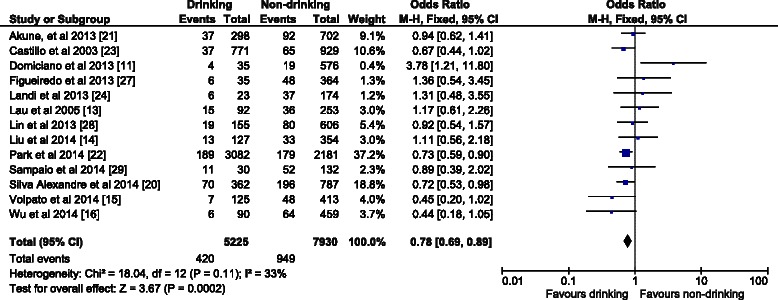
The forest plot of effect sizes for alcohol drinking overall population

The OR (95 % CI) in the fixed effect model for males (Fig. 2) was 0.67 (0.54–0.83); the overall effect was Z (6) = 3.67 (*p* = 0.0002), which was statistically significant. There was low heterogeneity (I^2^ = 0 %). For females the OR (95 % CI) in the fixed effect model (Fig. 3) was 0.92 (0.75–1.11). The overall effect was not significant Z (6) = 0.88 (*p* = 0.38); heterogeneity was moderate (I^2^ = 28 %). In contrast with the females’ analysis, the model for the overall population was robust. The OR (95 % CI) in the fixed effect model (Fig. 4) was 0.78 (0.69–0.89), the overall effect was Z (12) = 3.67 (*p* = 0.0002). Heterogeneity was moderate (I^2^ = 33 %). All the ORs were below 1, so the results suggest that alcohol consumption did not contribute to the risk of sarcopenia.

### Sensitivity analysis

A publication bias was not detected for data from alcohol drinking males. In addition, the results were well-balanced. OR (95 % CI) with both models having the same statistically significant value of 0.67 (0.54–0.83). The only difference was in the tests for overall effect using the fixed model, where Z (6) = 3.64 (*p* = 0.0003), and the random effect model, where Z (6) = 3.67 (*p* = 0.0002). Heterogeneity was low in both the cases (I^2^ = 0 %). In contrast to males, the results in females’ analysis were not as consistent. Heterogeneity was low; nevertheless, one study-Domiciano et al. [11] was excluded due to publication bias. The most robust result was in the fixed effect model after excluding that study. There the OR (95 % CI) was 0.89 (0.73–1.08), which was not statistically significant. Moreover, the test for the overall effect was very low level Z (5) = 1.16 (*p* = 0.25), I^2^ = 0 %. In the overall population, the fixed effect model excluding Domiciano et al. [11] was the strongest, with Z (11) = 3.86 (*p* = 0.0001), I^2^ = 0 %. The OR (95 % CI) was 0.77 (0.68–0.88).

### Summary of findings table

We used optimal ORs according to the sensitivity analyses to create a table summarizing the findings. The estimated risk of sarcopenia in the non-drinking males group was 128 per 1000 and the corresponding risk in the drinking males group was 89 per 1000 (95 % CI 73 to 108). The corresponding risk in medium risk population was 92 per 1000 (95 % CI 76 to 112) in the drinking males group. The estimated risk of sarcopenia in the non-drinking females group was 111 per 1000 and the corresponding risk in the drinking females group was 100 per 1000 (95 % CI 83 to 120). The corresponding risk in medium risk population was 86 per 1000 (95 % CI 72 to 104) in the drinking females group. The estimated risk in the non-drinking overall population was 122 per 1000 and the corresponding risk in the drinking females was 96 per 1000 (95 % CI 85 to 109). The corresponding risk in medium risk population was 93 per 1000 (95 % CI 82 to 105) the drinking overall population. The non-drinking group always had a higher proportion of people who were sarcopenic and excepting females the finding was statistically significant. Those analyses did not confirm that alcohol drinking is a risk factor for sarcopenia development; quite the opposite.

## Discussion

The results of the meta-analyses show that alcohol drinking did not contribute to sarcopenia development. The proportion of people categorized as sarcopenic was less, albeit not always significantly, in alcohol drinking group in all the analyses. This finding was strengthened by sensitivity analysis. Our findings notwithstanding, there were several problems encountered in the meta-analysis.

There were huge ambiguities in scientific approaches across the studies, for example, in diagnosing sarcopenia. Currently the EWGSOP algorithm [12] is considered the best tool for diagnosing sarcopenia. For the purposes of this meta-analyses, the EWGSOP algorithm and muscle mass measurement by dual-energy X-ray absorptiometry (DEXA) or bioelectrical impedance analysis (BIA) were considered suitable methods of sarcopenia diagnosis. Even though the EWGSOP has established cut off points for DEXA determined appendicular skeletal muscle mass (ASM) (i.e., 7.26 kg/m^2^ for men and 5.5 kg/m^2^ for women) and skeletal muscle index (SMI) (i.e., 7.23 or 7.25 kg/m^2^ for men and 5.67 kg/m^2^ for women), different cut off points for ASM or SMI were used in almost every study. For example, for males SMI cut off points of <5.72 kg/m^2^ [13] and <7.0 kg/m^2^ [14] were used [13, 14], for females cut off points of <4.82 kg/m^2^ < 5.9 kg/m^2^ were used [13, 14]. The EWGSOP has also established cut off points for muscle mass determined by BIA. Nevertheless, the SMIs cut off points used in the articles included in this review varied considerably. The SMI cut off points for males ranged from <7.0 kg/m^2^ [10] to <8.82 kg/m^2^ [15]; for females they ranged from <5.67 kg/m^2^ [16] to <6.42 kg/m^2^ [15]. This may be one reason the prevalence of sarcopenia varied so widely- from 3.8 % in the study by Domiciano et al. [11] to 27.2 % in the study by Lau et al. [13].

Variability and a lack of objectivity in the description of alcohol exposure might also have affected our results. It was almost impossible to find precise cumulative data such as a total number of alcohol units over time (e.g., drink-years). Exposure status was described using incompatible categories according to the daily amount, exposure period in the subjects’ lifetimes or current habits. Therefore, it was difficult in this work to find and establish an optimal stratification for exposure. In any event, all approaches were based on the subjective summarization by the participants. Although in recent years self-reports has been shown repeatedly to have good concordance with other methods [17], they can be influenced by deliberate under- or overestimation of consumption and by failures of memory and other cognitive factors [18]. Nevertheless, some suitable methods have been created previously, for example, the alcohol use disorders identification test (AUDIT) [19], following the recommendations of the World Health Organization (WHO). Unfortunately, AUDIT does not divide people into the categories of drinkers and nondrinkers. Anyway, the subjective component of alcohol exposure probably contributed to the huge variability of percentage of alcohol drinkers described in individual studies.

Despite problems with diagnosing sarcopenia and ascertaining exposure we detected a statistically significant age-difference between sarcopenia and no sarcopenia groups. That difference could work as important confounder of results. Aging is known to play a significant role in sarcopenia development. The mean age differed by more than 5 years in some of the studies we included. Such a difference existed in males studied by Liu et al. [14] and Silva Alexandre et al. [20], in females studied by Akune et al. [21] and Silva Alexandre et al. [15], and in the overall population studied Volpato et al. [15] and Wu et al. [16].

Beyond the aforementioned limitations, we must note that no study we examined had a primary focus on the relationship between sarcopenia and alcohol consumption. In all studies, alcohol consumption was merely one of many observed variables. Also, the exclusion and inclusion criteria varied between studies and resulted in the elimination of participants with characteristics which could be interesting to explore. Among individuals excluded from participation in different studies were those with poor functional status or metal implants [14] and those with limb edema, a pacemaker, joint prosthesis, or severe varicosities [15]. Other studies excluded older adults who answered yes for the question “Do you currently have kidney failure?” [22] or who were unable to perform the handgrip strength test or the walking portion of the Short Physical Performance Battery (SPPB), or who were unable to stand for measurement of weight and height [20]. Some of these exclusions might not be necessary in the event that the main aim of study was to explore the relation between sarcopenia and alcohol consumption. There were thus excluded subjects who were older, had less education, drank less, reported more difficulties in activities of daily living (ADL) and instrumental activities of daily living (IADL), had more sever hypertension, diabetes, lung disease, heart disease, stroke, falls, instances of hospitalization, a more sedentary lifestyle, more cognitive impairment, undernutrition and risk for undernutrition according to the Mini-nutritional assessment (MNA) [20]. Nevertheless, the inclusion of those subjects in the meta-analysis could confound the results. In contrast, exclusion criteria were not an issue is some studies [13, 21, 23, 24]. All the data from these studies were clustered into the analyses without regard to whether participants were accepted according to the same exclusion and inclusion criteria. Therefore, the cluster of the included studies could bring a little bit different results if the criteria were the same for all of them.

We also need to note that our results may have been influenced due to different design between cohort studies and cross-sectional studies. Excepting the results of Castillo et al. [21], the results of individual cohort studies indicated that for the overall population alcohol consumption might increase the risk of sarcopenia, which was completely opposite to the results in cross-sectional studies. However, the design of those studies was classified by their authors. We accepted their classifications in good faith even though there could be some concerns that the classifications might not have been exactly correct. Epidemiological studies can be truly cross-sectional (present drinking and present sarcopenia), case control (drinking history and present sarcopenia), or cohort (present drinking and future sarcopenia). Moreover, out of cohort studies we only used the data from baselines, therefore they could be considered as case–control studies rather than cohort studies and as such they do provide a higher level of evidence than cross-sectional studies. Consolidation of the two study designs could be justified in this meta-analysis, because they both are observational studies and they all were based on a similar approach in extracting information about alcohol consumption and sarcopenia diagnostics.

The results of the study show that alcohol consumption is not associated with sarcopenia development. Boffetta and Garfinkel [25] came to a similar conclusion regarding alcohol consumption and mortality and coronary heart disease (CHD). There, moderate alcohol intake had a protective effect on CHD mortality. Other authors have partly confirmed the possible protective influence of alcohol consumption on CHD mortality [26].

To sum up, we found out that alcohol consumption was not a risk factor for the development of sarcopenia, even more, according to the results alcohol consumption could have protective character against sarcopenia. This does not mean, however, that the authors recommend drinking alcohol to prevent any diseases.

## Conclusion

The implications for research would be summarized into three recommendations. First, it would be beneficial to diagnose sarcopenia more specifically using age, gender and ethnicity specific reference values. Second, valid tools for quantifying exposure need to be developed and employed. Finally, it would be useful to study groups more similar according to the age. Those groups’ comparison would be much more meaningful.

### Ethics approval and consent to participate

Not applicable.

### Consent for publication

Not applicable.

### Availability of data and materials

Not applicable: As a meta-analysis, the data gathered from other sources may or may not be publicly available or in a repository.

## Abbreviations

ADL: activities of daily living
ASM: appendicular skeletal muscle mass
AUDIT: alcohol use disorders identification test
BIA: bioelectrical impedance analysis
CHD: coronary heart disease
DEXA: dual-energy X-ray absorptiometry
EWGSOP: European working group on sarcopenia in older people
IADL: instrumental activities of daily living
MNA: mini-nutritional assessment
OR: odds ratio
SMI: skeletal muscle index
SPPB: short physical performance battery
WHO: World Health Organization
